# Screening colorectal cancer associated autoantigens through multi-omics analysis and diagnostic performance evaluation of corresponding autoantibodies

**DOI:** 10.1186/s12885-025-14080-5

**Published:** 2025-04-16

**Authors:** Zan Qiu, Yifan Cheng, Haiyan Liu, Tiandong Li, Yinan Jiang, Yin Lu, Donglin Jiang, Xiaoyue Zhang, Xinwei Wang, Zirui Kang, Lei Peng, Keyan Wang, Liping Dai, Hua Ye, Peng Wang, Jianxiang Shi

**Affiliations:** 1 State Key Laboratory of Metabolic Dysregulation & Prevention and Treatment of Esophageal Cancer, Henan Institute of Medical and Pharmaceutical Sciences, Zhengzhou University, Zhengzhou, 450052 Henan China; 2 College of Public Health, Zhengzhou University, Henan 450001 Zhengzhou, China; 3 Henan Key Laboratory of Tumor Epidemiology, Zhengzhou University, Zhengzhou, 450052 Henan China; 4 Division of Pediatric Surgery, Department of Surgery, Children’s Hospital of Pittsburgh, University of Pittsburgh School of Medicine, PA 15224 Pittsburgh, USA

**Keywords:** Colorectal cancer, Immunodiagnostic, Tumor-associated autoantibodies, Multi-omics, Machine learning

## Abstract

**Background:**

This study aims to screen, validate novel biomarkers and develop a user-friendly online tool for the detection of colorectal cancer (CRC).

**Methods:**

Multi-omics approach, comprising proteomic analysis and single-cell transcriptomic analysis, was utilized to discover candidate tumor-associated antigens (TAAs). The presence of tumor-associated autoantibodies (TAAbs) in serum was subsequently assessed using enzyme-linked immunosorbent assays (ELISA) in 300 CRC patients and 300 healthy controls. Ten machine learning algorithms were utilized to develop diagnostic models, with the optimal one selected and integrated into an R Shiny-based GUI to enhance usability and accessibility.

**Results:**

We identified twelve potential TAAs: HMGA1, NPM1, EIF1AX, CKS1B, HSP90AB1, ACTG1, S100A11, maspin, ANXA3, eEF2, P4HB, and HKDC1. ELISA results showed that five TAAbs including anti-CKS1B, anti-S100A11, anti-maspin, anti-ANXA3, and anti-eEF2 were potential diagnostic biomarkers during the diagnostic evaluation phase (all *P* < 0.05). The Random Forest model yielded an AUC of 0.82 (95% CI: 0.78–0.88) on the training set and 0.75 (95% CI: 0.68–0.82) on the test set, demonstrating the robustness of the results. Web-based implementations of CRC diagnostic tools are publicly accessible via weblink https://qzan.shinyapps.io/CRCPred/.

**Conclusions:**

A five biomarker panel can server as complementary biomarker to CEA and CA19-9 in CRC detection.

**Supplementary Information:**

The online version contains supplementary material available at 10.1186/s12885-025-14080-5.

## Introduction

Colorectal cancer (CRC) is the third most commonly diagnosed cancer worldwide and the second leading contributor to cancer-related mortality [1]. While CRC predominantly affects individuals over 50 years, a notable increase in incidence among younger populations has been reported [2]. Due to the poor prognosis associated with advanced-stage CRC, where five-year survival rates drop below 15%, early detection through regular screening programs is critical [3–5]. In clinical practice, sigmoidoscopy and colonoscopy are currently the standard tests for CRC diagnosis because of their high sensitivity and ability to detect visible precancerous lesions [6]. Additionally, stool-based tests can increase the efficiency of colonoscopy utilization [7]. Commercially available fecal immunochemical test shows a moderate sensitivity of 67.3% for CRC detection, while the multi-target stool DNA test demonstrates superior diagnostic performance with a sensitivity of 93.9% [8]. Although methylated SEPT9 is the only FDA-approved blood-based biomarker for CRC screening, its clinical utility is hampered by limited sensitivity, detecting only 44.7% of early-stage CRC and 11.2% of advanced adenomas [9]. Conventional clinical tumor markers, such as CEA and CA19-9, shows limited sensitivity, furtherly highlighting the potential need for more efficient and patient-friendly diagnosis options [10–12].

Tumor-associated autoantibodies (TAAb) have attracted attention as potential biomarkers for cancer diagnosis due to their stable presence in the bloodstream, even when corresponding antigen levels are low [13]. Autoantibodies can be detected earlier than the clinical onset of cancer, highlighting their value for early diagnosis [14]. Individual TAAbs have limited sensitivity and specificity, necessitating the combination of numerous TAAbs to increase diagnostic accuracy [15]. Anti‑p53 antibodies, the most extensively researched autoantibodies in CRC, may serve as biomarkers to distinguish CRC from healthy individuals or benign patients, a potential supported by a summary receiver operating characteristic curve with an AUC of 0.78 (95% CI: 0.76–0.81) [16].

However, the significance of identifying new tumor-associated antigens (TAAs) cannot be neglected, as autoantibodies, antibodies that target self-antigens, play a crucial role in modulating inflammatory responses, maintaining immune system homeostasis, and distinguishing between normal and tumor individuals in certain contexts [17]. In previous studies, the utilization of various techniques such as serological analysis of recombinant tumor cDNA expression libraries [18], phage cDNA libraries [19], serological proteome analysis(SERPA) [20], and protein microarrays [21] for TAAs identification in CRC.

Data mining is a valuable tool for identifying potentially useful patterns within large datasets, providing a more precise and reliable estimate of the efficiency of autoantibodies in CRC detection [14, 16]. The consensus molecular subtypes (CMS) of CRC, defined by integrating multi-omics data including genomics, epigenomics, transcriptomics, and immune-related proteomics, provide a comprehensive classification system that enables the identification of molecular markers with broad generalizability for CRC diagnosis [22]. Proteomic analyses elucidate distinct protein expression profiles, and previous studies also provide data for each CMS subtype [23]. Moreover, single-cell transcriptomics allows for a comprehensive analysis of the heterogeneity of CRC and modifications within the immune microenvironment at the single-cell level, facilitating the investigation of potential changes originating from epithelial cells [24, 25]. The integration of multi-omics approach facilitates the identification of novel TAAs, thereby providing a more comprehensive foundation for CRC diagnosis [26].

Our study aims to identify TAAs by using proteomic and single cell transcriptomic analysis, evaluate the diagnostic performance of their corresponding autoantibodies, and provide a scalable, cost-effective, and minimally invasive alternative to facilitate the detection of CRC.

## Materials and methods

### Participating patients and sample collection

This study included two groups: 300 CRC patients as CRC group, and 300 healthy controls (HCs) as HC group. Participants were matched by age (± 5 years) and gender, and were randomized in a 7:3 ratio, divided into a training set and a test set. The serum samples used in the study were from the Biological Specimen Bank of Henan Key Laboratory of Tumor Epidemiology (Henan, China) spanned from October 2020 to December 2023. All enrolled primary CRC cases were verified through pathological examination and were treatment-naïve. HCs were confirmed by reviewing their medical records to ensure they were free from malignancies or immune-related diseases. This study was approved by the Institutional Review Board of Zhengzhou University (Approval number: ZZURIB 2019-002). Written informed consent forms were obtained from all participants. All procedures were conducted in accordance with the relevant guidelines and regulations, as well as the Declaration of Helsinki. Early stages were categorized as stages 0 through II, and late stages were categorized as stages III and IV.

The blood samples were centrifuged at 3000 g for 5 min and the serum were aliquoted for long time storage in -80℃ freezer.

### Identification of candidate TAAs based on multi-omics

The single-cell transcriptome data in the study were obtained from the Gene Expression Omnibus (GEO) database, including GSE132465, GSE144735 [24] and GSE200997 [27]. The proteomic data of COAD were downloaded from the Proteomic database from the Clinical Proteomic Tumor Analysis Consortium (CPTAC) database [23]. Tumor and normal epithelial cells were compared to identify abnormally highly expressed genes. Proteomic data were used to verify the overexpression of the identified genes. Participants’ information of relevant studies is shown in Supplementary Table S1.

### Single-cell transcriptome data analysis

The R package Seurat (v4.3.2) was utilized to convert the matrix count for a single sample [28]. Subsequently, genes expressed in fewer than three cells were removed. Low-quality cells were eliminated based on the following criteria: cells containing fewer than 200 expressed genes, an erythrocyte ratio exceeding 10%, or mitochondrial content above 20%. Other processes are standard procedures [28]. Bulk effect correction is performed using harmony during the integration of three data sets [29]. Cell identity annotations for individual clusters are specified based on the expression of established marker genes and verified using CellTypist [30]. Subclusters of cells with comparable gene expression profiles are then assigned to the same cell type.

### Differential expression analysis for single cell transcriptomic data

The wilcox.test algorithm within the FindMarkers function of the Seurat package was employed to identify differentially expressed genes in epithelial cells, using thresholds of Log₂FC > 0.25 and adj.*P* < 0.05. Subsequently, the subset function was utilized to segregate cells based on CMS classification. The same criteria of Log₂FC > 0.25 and adj.*P* < 0.05 were applied to obtain up-regulated differentially expressed genes across various CMS subtypes.

### Differential expression analysis for proteomic data

The log-ratio normalized proteomic data were directly downloaded from the CPTAC database. Differentially expressed proteins were screened with log_2_FC > 0.6 and adj.*P* < 0.05 using limma package for differential analysis [31].

### Additional screening strategies are used to narrow down the candidate TAAs

Driver genes were collected from the IntOGen(CRC) [32] and OncoKB [33] databases. Genes within the CTDatabase [34] are named CT-related. A fetal gene expression signature [35–37] is supported by relevant articles, termed as Fetal_related.

In a separate study, the proteomic screening criteria were set at adj.*P* < 0.05 and log_2_FC > 0.3. The down-regulated genes in small intestine carcinoma (SBA) epithelial cells compared with controls intersected with up-regulated genes in CRC epithelial cells compared with controls were designated as BSA_related.

All the genes listed above can be found in the Supplementary Table S2.

### Function analysis of differentially expressed genes or proteins

Gene ontology (GO) annotation was performed to better understand the biological functions of these differentially expressed proteins. GO over-representation analysis of the selected genes was performed by using the clusterProfiler package [38].

### Recombinant proteins and the detection of TAAbs by ELISA

Eight proteins (NPM1, EIF1AX, CKS1B, eEF2 (encoded by *EEF2*), P4HB, ANXA3, S100A11, HSP90AB1) were purchased from CUSABIO (Wuhan, China), and four proteins (HMGA1, ACTG1, maspin (encoded by *SERPINB5*), HKDC1) were purchased from Cloud-clone Corporation (Wuhan, China). The concentration, purity, and molecular weight of all proteins were confirmed using SDS/PAGE gel. The enzyme linked immunosorbent assay was performed with a coating concentration of 0.125 ug/ml for HMGA1, NPM1, S100A11 and 0.25 ug/ml for EIF1AX, HSP90AB1, ACTG1, CKS1B, maspin, ANXA3, eEF2, P4HB, HKDC1. The ELISA procedures were described in our previous study [39, 40]. Specific Binding Index (SBI) was used to evaluate the level of autoantibodies in peripheral serum, which represents the degree of binding between antigen and antibody. SBI = (OD_TBD_ - OD_Blank_) / (OD_QC_ - OD_Blank_). OD_TBD_ refers to the optical density (OD) value that needs to be determined, OD_QC_ represents the average OD value of the quality control (QC) samples, and OD_Blank_ denotes the average OD value of the blank wells.

### Diagnostic model development

Ten machine learning algorithms were employed using the “tidymodels” R package in the training set based on the SBI values of the five TAAbs. These models include Logistic Regression, Decision Tree, Elastic Net, K-Nearest Neighbours, Light Gradient Boosting Machine, Random Forest (RF), eXtreme Gradient Boosting, Support Vector Machine, Multilayer Perceptron via nnet, and Stacking ensemble, chosen for their diverse methodologies and robust performance in identifying complex patterns within the data. A 10-fold cross-validation was performed to evaluate the predictive ability of the model. Precision-recall (PR) curves were employed to evaluate the ability of the model to discriminate, and decision curve analysis (DCA) were used to confirm the clinical effectiveness of the model further. Model performance was compared using AUCs on both training and test datasets. Statistical significance between datasets was evaluated using DeLong tests. The same statistical method was applied to assess differences in AUCs between models with equivalent sample sizes.

### Statistical analysis

Data analysis and visualization was performed using SPSS Statistics 26.0 and R-4.3.2 software. Sample size calculation was performed using PASS software (Version 15, Confidence Intervals for One Proportion). Based on this analysis, the minimum sample size of the test set used in model development is 85 CRC patients and 58 HC participants. ROC analysis and the AUC with 95% CI were used to evaluate the diagnostic performance of the biomarkers and the model. The sensitivity and specificity were determined based on the cutoff value, which was defined as the SBI value at the maximum Youden index, while specificity is more than 85%. If either the TAAbs model or the clinical biomarker test yields a positive outcome, the individual is classified as positive. The corresponding positivity rate is then calculated. Three components chi-squared test is used to compare the diagnostic performance by using SPSS. Specifically, single-cell plotting is carried out using scRNAtoolVis (https://github.com/junjunlab/scRNAtoolVis) and plot1cell [41]. Analyses were judged statistically significant with a two-sided *P*-value of < 0.05.

## Results

### Study design and sample characteristics

**Fig. 1:**
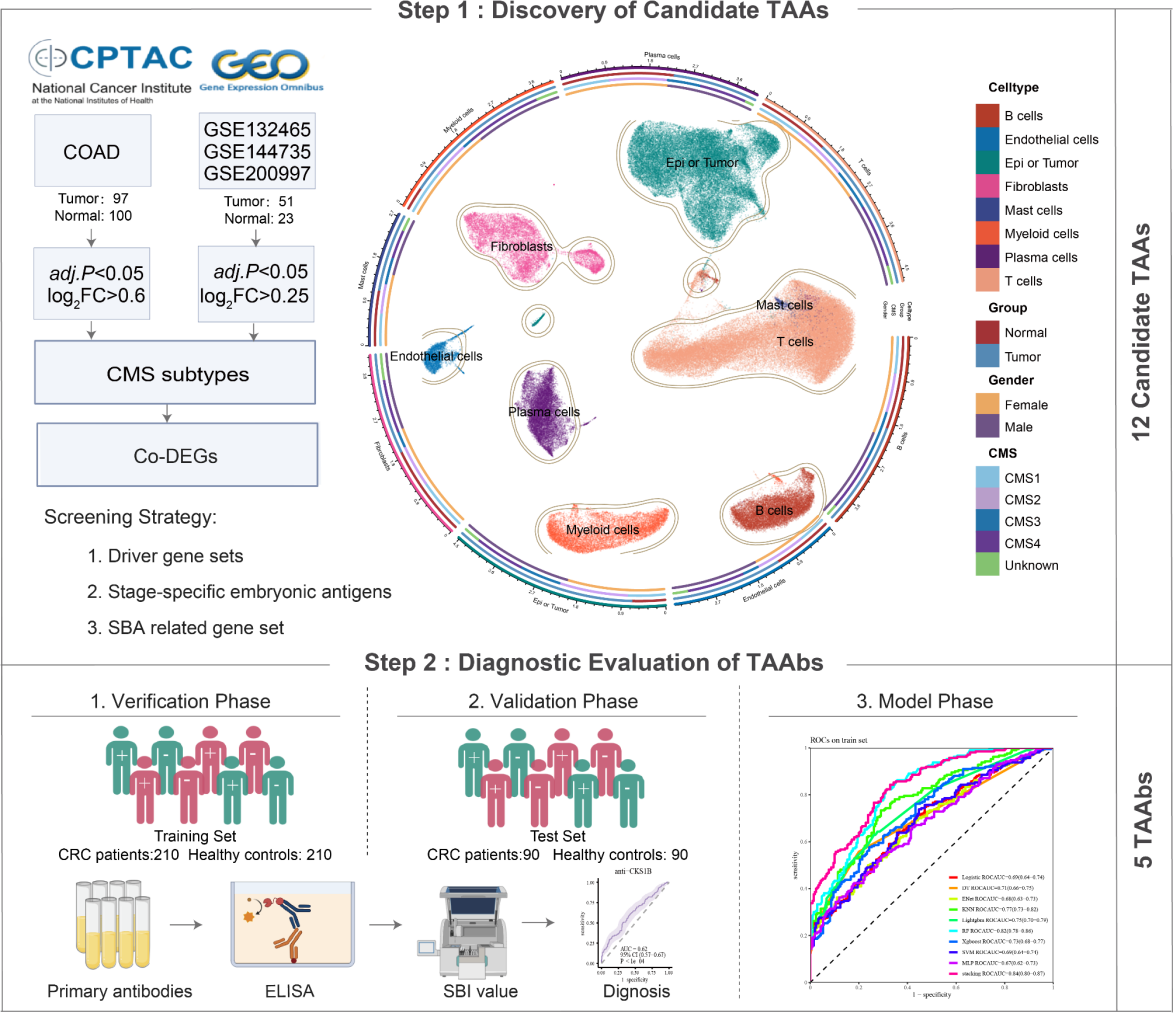
The flow diagram of this study. This study was conducted in two steps: discovery of candidate TAAs (Step 1) and evaluation of TAAbs (Step 2). TAAs Tumor-associated antigens, TAAbs Tumor-associated autoantibodies, CMS Consensus Molecular Subtypes, DEG Differential Expression Analysis, ELISA Enzyme-linked immunosorbent assay, SBA Small Bowel Adenocarcinoma, SBI Specific Binding Index

This study was conducted in two steps: discovery of candidate TAAs (Step 1) and evaluation of TAAbs (Step 2) (Fig. 1). Proteomic data from 100 normal controls and 97 tumor cases were included in current study, and single-cell transcriptome data from 23 normal controls and 51 tumor cases. More detailed information can be found in Supplementary Table S1. A primary CRC single-cell transcriptome atlas was constructed, revealing an increased percentage of epithelial cells. Candidate TAAs were selected using screening strategies as shown in Fig. 1.

Subsequently, serum autoantibodies against 12 candidate TAAs were further evaluated by ELISA. The verification and validation phases were employed to assess the diagnostic performance of TAAbs. During the verification phase, diagnostic models were constructed in the training set, which included 210 CRC cases and 210 HC participants. In the validation phase, a test set comprising 90 CRC cases and 90 HC individuals was utilized to verify the potential diagnostic value of these TAAbs. Demographic and clinical characteristics of the study participants are presented in Table 1. Majority patients were diagnosed with stage II CRC in this study, including 118 (56.2%) in training set and 53 (58.9%) in test set. In accordance with routine clinical practice, CEA and CA19-9 were considered elevated above cutoff values of 5 ng/ml and 35 ng/ml, respectively. In both the training and test sets, the positive rates for CEA were 31.2% and 32.2%, while those for CA19-9 were 18.6% and 12.2%, respectively. In the model phase, 10 machine learning models were used for model selection and training, and hyperparameter tuning was used to improve the performance of the mode.

**Table 1:** Baseline characteristics of participants in the training set and test set

Characteristics	Training Set		*P*	Test Set		*P*
	CRC (*n* = 210)	HC (*n* = 210)		CRC (*n* = 90)	HC (*n* = 90)	
**Ages(years)**			0.574			0.735
Mean ± SD	58.8 ± 13.4	59.2 ± 14.2		60.0 ± 13.8	58.9 ± 12.9	
**Gender**,* n***(%)**			0.225			1
Male	108 (51.4)	113 (53.8)		56 (62.2)	51 (56.7)	
Female	102 (48.6)	97 (46.2)		34 (37.8)	39 (43.3)	
**Site**,* n***(%)**						
Colon	73 (34.8)			39 (43.3)		
Rectum	118 (56.2)			44 (48.9)		
Unknow	19 (9.0)			7 (7.8)		
**TNM stage**,* n***(%)**						
0-I	23 (11.0)			8 (8.9)		
II	118 (56.2)			53 (58.9)		
III	11 (5.2)			5 (5.6)		
**Unknow**	58 (27.6)			24 (26.7)		
**CEA**,** ng/ml**,* n***(%)**						
**< 5**	116(55.2)			56(62.2)		
**≥ 5**	76(31.2)			29(32.2)		
**Unknow**	18(8.6)			5(5.6)		
**CA19-9**,** ng/ml**,* n***(%)**						
**< 35**	154(73.3)			74(82.2)		
**≥ 35**	39(18.6)			11(12.2)		
**Unknow**	17(8.1)			5(5.6)		

### Identification of candidate TAAs based on multi-omics

**Fig. 2:**
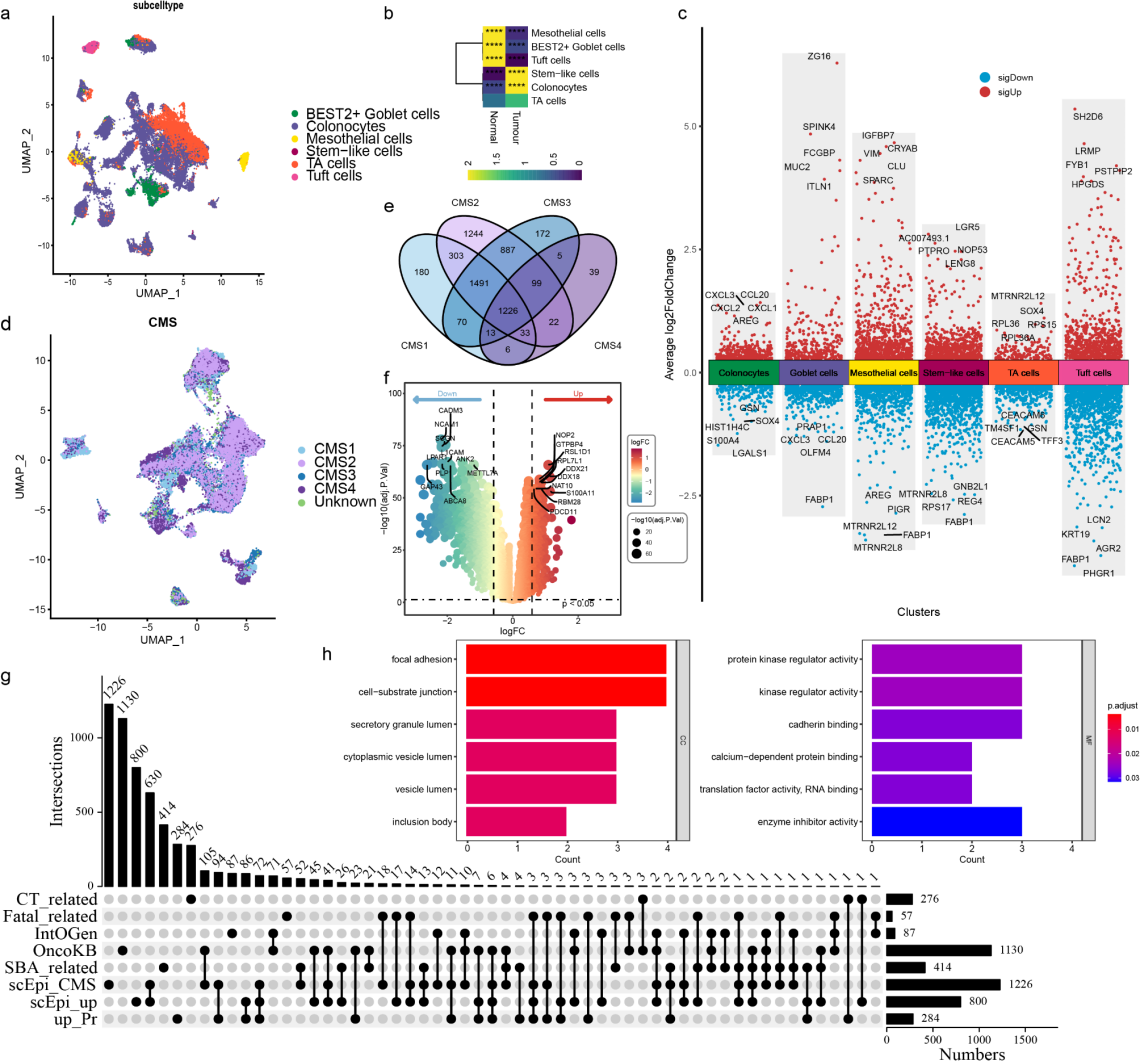
Identification of 12 candidate TAAbs based on multi-omics approach. **a** UMAP plot of CRC epithelial cells, color-coded by subcelltype subtype. **b** Heatmap showing the tissue preference of each cell subset as indicated by the odds ratios. **c** Volcano plot showing significantly upregulated or downregulated genes in each cluster, with the top five markers for each cluster highlighted. **d** UMAP plot of CRC epithelial cells, color-coded by CMS subtypes. **e** A four-set venn diagram showing the intersection of four CMS subtypes. **f** Volcano plot of the differentially expressed proteins between CRC and NC groups. **g** Upset plot for signature genes from different studies. **h** GO pathway enrichment analysis of 12 candidate TAAs

Epithelial cells were identified using markers (*EPCAM* and *KRT19*), and the subtypes of the epithelial cells were defined based on the reference map from CellTypist [42] (Fig. 2a). Odds ratios results showed that stem-like cells and colonocytes were enriched in tumor compared to adjacent controls (Fig. 2b). Stem-like cells showed upregulated *LGR5*, which is consistent with the accepted histological model of intestinal epithelium [43]. Compared to adjacent normal cells, tumor colonocytes overexpressed chemokines (*CXCL1*, *CXCL2*, *CXCL3*, and *CCL20*), showing significant effects on inflammatory processes and immune cell recruitment (Fig. 2c). Eight hundred upregulated genes were identified in epithelial cells for all CMS subtype combined. Furthermore, 1,226 genes over-expressed across four CMS subtypes were identified (Fig. 2d and e). Two hundred and eighty-four up-regulated proteins were identified from proteomic data (Fig. 2f).

As shown in Figs. 2g and 72 genes were thought to play an important role in the development of CRC. Further intersecting with gene sets of interest (listed in Supplementary Table S2) in TAAs findings, six driver genes in OncoKB and three genes from a fetal gene-expression signature were identified. In addition, *EEF2*, *P4HB* and *HKDC1* were derived from SBA-related genes screened by slightly different strategies.

Finally, twelve potential TAAs, derived from the proteins encoded by the genes *HMGA1*, *NPM1*, *EIF1AX*, *CKS1B*, *HSP90AB1*, *ACTG1*, *S100A11*, *SERPINB5*, *ANXA3*, *EEF2*, *P4HB*, and *HKDC1*, were selected for subsequent experimental validation and verification (Supplementary Table S3). These genes are enriched in pathways closely related to cancer initiation and progression (Fig. 2h).

### Diagnostic performance in the verification phase and validation phase

**Fig. 3:**
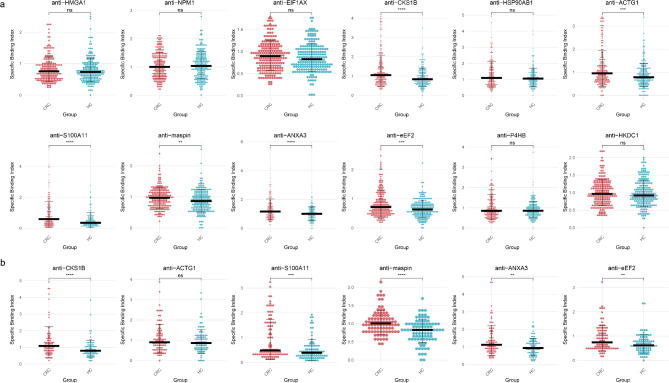
Serum autoantibody levels against candidate TAAs. **a** Scatter plot of the SBI values of the 12 candidate TAAbs in the training set. **b** Scatter plot of the SBI values of the 6 candidate TAAbs in the test set. *: *P* < 0.05, **: *P* < 0.01, ****: *P* < 0.0001, ns: *P* > 0.05

**Fig. 4:**
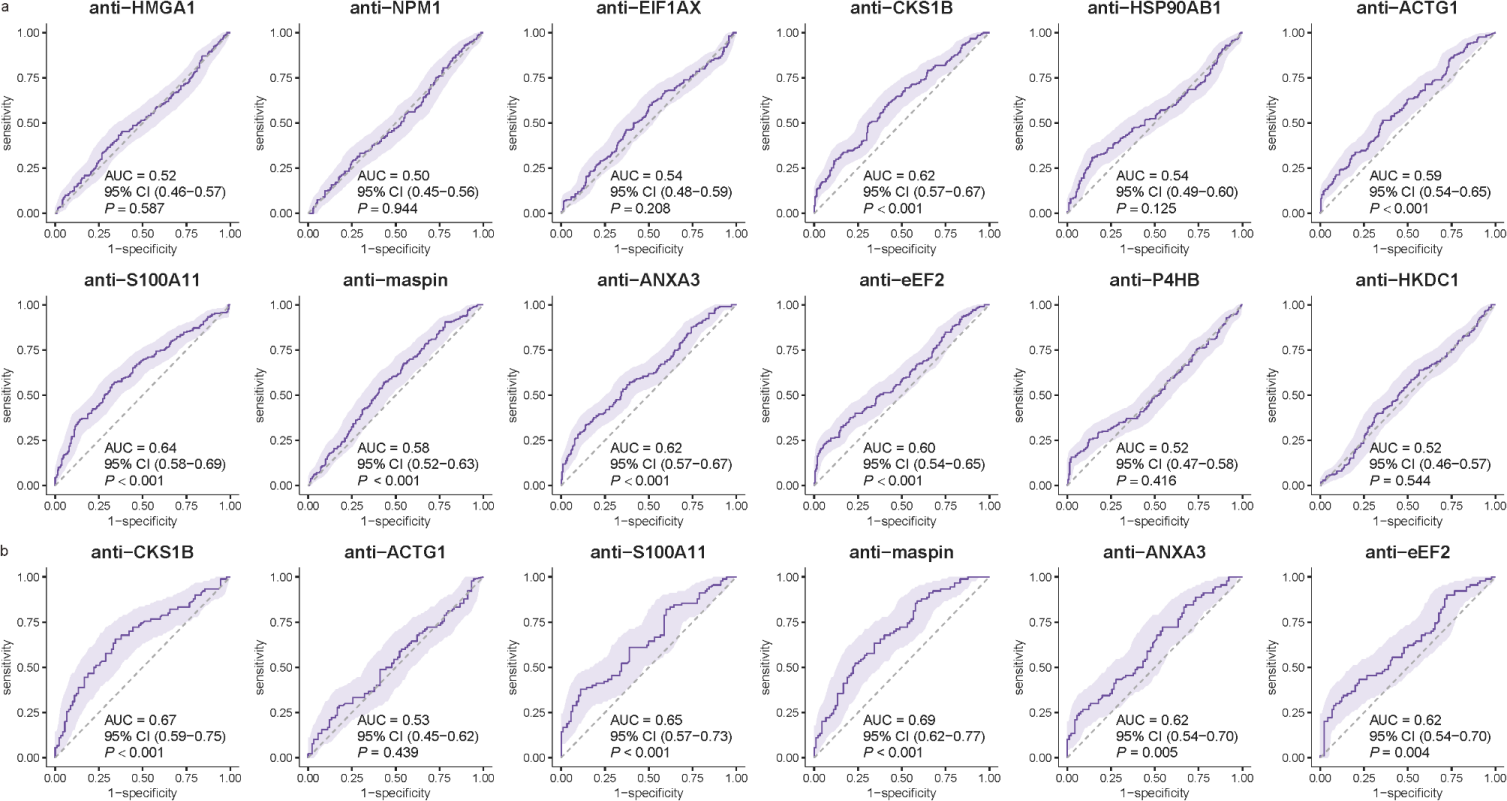
Diagnostic performance of the candidate TAAbs. **a** ROC curve of the 12 candidate TAAbs in the training set. **b** ROC curves of the 6 candidate TAAbs in the test set

In the verification phase, six TAAbs, including anti-CKS1B, anti-ACTG1, anti-S100A11, anti-maspin, anti-ANXA3, and anti-eEF2, showed significant differences between CRC patients and NC (all *P* < 0.05) (Fig. 3a). The AUC values ranged from 0.58 to 0.64. Specifically, the AUC values were as follows: anti-CKS1B (AUC = 0.62, 95% CI:0.57–0.67, *P *< 0.01), anti-ACTG1 (AUC = 0.59, 95% CI:0.54–0.65, *P *< 0.01), anti-S100A11 (AUC = 0.64, 95% CI:0.58–0.69, *P *< 0.01), anti-maspin (AUC = 0.58, 95% CI:0.52–0.63, *P *< 0.01), anti-ANXA3 (AUC = 0.62, 95% CI:0.57–0.67, *P *< 0.01), and anti-eEF2 (AUC = 0.60, 95% CI:0.54–0.65, *P *< 0.01) (Fig. 4a).

During validation, the diagnostic performance of six significant TAAbs from the training set was further assessed, with five of them demonstrating potential diagnostic value. The results showed that the AUCs for these TAAbs ranged from 0.53 to 0.69, the sensitivity ranged from 22.22% to 37.78%, and the specificity ranged from 86.67% to 91.11% (Table 2). Among them, anti-maspin exhibited the highest diagnostic potential with an AUC of 0.69(95% CI: 0.62–0.77), a sensitivity of 35.56%, and a specificity of 86.67%. Anti-ACTG1 exhibited the lowest AUC of 0.53 (95% CI: 0.45–0.62) for CRC (*P* = 0.439) (Fig. 4b).

**Table 2:** Diagnostic performance of the 12 candidate TAAbs

Data	TAAbs	AUC (95%CI)	Sen (%)	Spe (%)	+LR	-LR	YI	*P*
Training Set	Anti-HMGA1	0.52(0.46–0.57)	10.00	94.29	1.75	0.95	0.04	0.587
	Anti-NPM1	0.50(0.45–0.56)	12.38	90.48	1.30	0.97	0.03	0.944
	Anti-EIF1AX	0.54(0.48–0.59)	20.48	85.71	1.43	0.93	0.06	0.208
	Anti-CKS1B	0.62(0.57–0.67)	29.05	88.57	2.54	0.80	0.18	< 0.001
	Anti-HSP90AB1	0.54(0.49–0.60)	30.95	85.71	2.17	0.81	0.17	0.125
	Anti-ACTG1	0.59(0.54–0.65)	24.29	88.57	2.12	0.85	0.13	< 0.001
	Anti-S100A11	0.64(0.58–0.69)	36.67	85.71	2.57	0.74	0.22	< 0.001
	Anti-maspin	0.58(0.52–0.63)	20.48	85.24	1.39	0.93	0.06	< 0.001
	Anti-ANXA3	0.62(0.57–0.67)	33.33	86.19	2.41	0.77	0.20	< 0.001
	Anti-eEF2	0.60(0.54–0.65)	22.86	94.29	4.00	0.82	0.17	< 0.001
	Anti-P4HB	0.52(0.47–0.58)	15.71	97.62	6.60	0.86	0.13	0.416
	Anti-HKDC1	0.52(0.46–0.57)	5.24	96.19	1.38	0.99	0.01	0.544
Test Set	Anti-CKS1B	0.67(0.59–0.75)	34.44	88.89	3.10	0.74	0.23	< 0.001
	Anti-ACTG1	0.53(0.45–0.62)	22.22	86.67	1.67	0.90	0.09	0.439
	Anti-S100A11	0.65(0.57–0.73)	37.78	88.89	3.40	0.70	0.27	< 0.001
	Anti-maspin	0.69(0.62–0.77)	35.56	86.67	2.67	0.74	0.22	< 0.001
	Anti-ANXA3	0.62(0.54–0.70)	26.67	91.11	3.00	0.80	0.18	0.005
	Anti-eEF2	0.62(0.54–0.70)	34.44	86.67	2.58	0.76	0.21	0.004

Sen Sensitivity, Sep Specificity, +LR Positive Likelihood Ratio, -LR Negative Likelihood Ratio, YI Youden Index

The results from the validation phase were consistent with those from the verification phase except for the autoantibody against ACTG1 (Fig. 3b). Consequently, data from the five TAAbs: anti-CKS1B, anti-S100A11, anti-maspin, anti-ANXA3, and anti-eEF2 were used for diagnostic model construction. There is no strong correlation between the relevant indicators in both the training and test sets.

### Diagnostic performance of the immunodiagnostic model based on machine learning

**Fig. 5:**
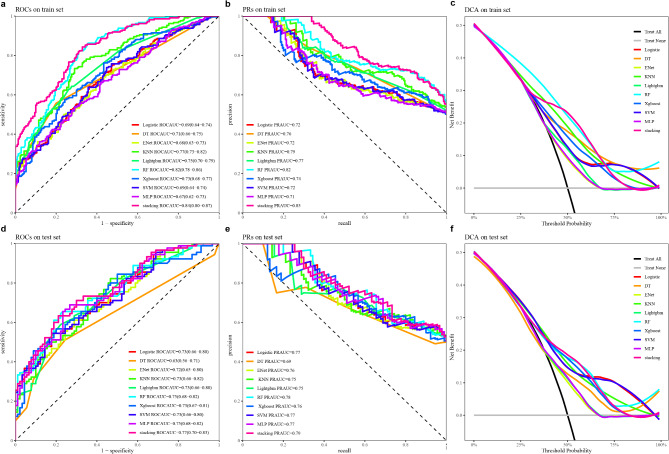
Comparative performance evaluation of 10 machine learning models for CRC diagnosis. **a** ROC curve analysis demonstrating the diagnostic ability associated with different models in the training set. **b** PR curve analysis demonstrating the ability to distinguish associated with different models in the training set. **c** DCA demonstrating the net benefit associated with different models in the training set. **d** ROC curve analysis demonstrating the diagnostic ability associated with different models in the test set. **e** PR curve analysis demonstrating the ability to distinguish associated with different models in the test set. **f** DCA demonstrating the net benefit associated with different models in the test set. LR Logistic Regression, DT Decision Tree, Enet Elastic Net, KNN K-Nearest Neighbors, LightGBM Light Gradient Boosting Machine, RF random forest, Xgboost eXtreme Gradient Boosting, SVM Support Vector Machine, MLP Multilayer Perceptron via nnet

The AUCs of ten models in the training set varied from 0.67 to 0.84, and their accuracy ranged from 61.90 to 72.62% (Fig. 5a; Table 3). Similarly, in the test set, the AUC ranged from 0.63 to 0.77 and the accuracy ranged from 61.11 to 68.33% (Fig. 5d; Table 3). The DeLong test showed no difference in AUCs between the training set and test sets for each model (Table 3).

**Table 3:** Diagnostic performance of the 10 machine learning algorithms in the training and test sets

Model	Data	AUC	95%CI	Sen(%)	Sep(%)	PPV(%)	NPV(%)	ACC(%)	F1	YI	Kappa	*P*
LR	Training Set	0.69	0.64–0.74	26.19	97.62	91.67	56.94	61.90	0.41	0.24	0.24	0.338
	Test Set	0.73	0.66–0.80	31.11	96.67	90.32	58.39	63.89	0.46	0.28	0.28	
DT	Training Set	0.71	0.66–0.75	32.38	93.81	83.95	58.11	63.10	0.47	0.26	0.26	0.105
	Test Set	0.64	0.56–0.71	31.11	91.11	77.78	56.94	61.11	0.44	0.22	0.22	
ENet	Training Set	0.68	0.63–0.73	36.19	89.05	76.77	58.26	62.62	0.49	0.25	0.25	0.357
	Test Set	0.72	0.65–0.80	38.89	91.11	81.4	59.85	65.00	0.53	0.30	0.30	
KNN	Training Set	0.77	0.73–0.82	48.1	87.62	79.53	62.80	67.86	0.60	0.36	0.36	0.361
	Test Set	0.73	0.66–0.81	46.67	85.56	76.36	61.60	66.11	0.58	0.32	0.32	
Lightgbm	Training Set	0.75	0.70–0.79	46.67	86.19	77.17	61.77	66.43	0.58	0.33	0.33	0.674
	Test Set	0.73	0.66–0.80	48.89	85.56	77.19	62.60	67.22	0.60	0.34	0.34	
RF	Training Set	0.82	0.78–0.86	50.95	85.24	77.54	63.48	68.10	0.61	0.36	0.36	0.078
	Test Set	0.75	0.68–0.82	51.11	83.33	75.41	63.03	67.22	0.61	0.34	0.34	
Xgboost	Training Set	0.73	0.68–0.77	42.86	87.14	76.92	60.40	65.00	0.55	0.30	0.30	0.830
	Test Set	0.74	0.66–0.81	45.56	88.89	80.39	62.02	67.22	0.58	0.34	0.34	
SVM	Training Set	0.69	0.64–0.74	27.62	96.19	87.88	57.06	61.90	0.42	0.24	0.24	0.351
	Test Set	0.73	0.66–0.80	32.22	96.67	90.63	58.78	64.44	0.48	0.29	0.29	
MLP	Training Set	0.67	0.62–0.73	36.19	88.10	75.25	57.99	62.14	0.49	0.24	0.24	0.089
	Test Set	0.75	0.68–0.82	38.89	91.11	81.40	59.85	65.00	0.53	0.30	0.30	
Stacking	Training Set	0.84	0.80–0.87	55.71	89.52	84.17	66.90	72.62	0.67	0.45	0.45	0.068
	Test Set	0.77	0.70–0.83	52.22	84.44	77.05	63.87	68.33	0.62	0.37	0.37	

LR Logistic Regression, DT Decision Tree, Enet Elastic Net, KNN K-Nearest Neighbors, LightGBM Light Gradient Boosting Machine, RF random forest, Xgboost eXtreme Gradient Boosting, SVM Support Vector Machine, MLP Multilayer Perceptron via nnet, Sen Sensitivity, Sep Specificity, PPV Positive Predictive Value, NPV Negative predictive value, ACC Accuracy, YI Youden Index

The stacking model showed the optimal diagnostic performance in the training set (AUC: 0.84, 95%CI = 0.80–0.87), followed by the RF model (AUC: 0.82, 95%CI = 0.78–0.86) (Fig. 5a and d). The DeLong test showed no significant difference between the two models. Additionally, the PR curve shows that the stacking model is better than the RF model. (Figure 5b and e). But DCA indicated the clinical effectiveness of the RF model in both the training set and the test set (Fig. 5c and f). To ensure a robust comparison of the RF and Stacking models, we evaluated their performance on both the training and test datasets using Delong test. In the test set, the AUC comparison showed *P* = 0.204. This suggests that both the RF and Stacking models exhibit comparable generalization performance on test data. Therefore, the simpler RF model is more suitable for clinical diagnosis.

**Fig. 6:**
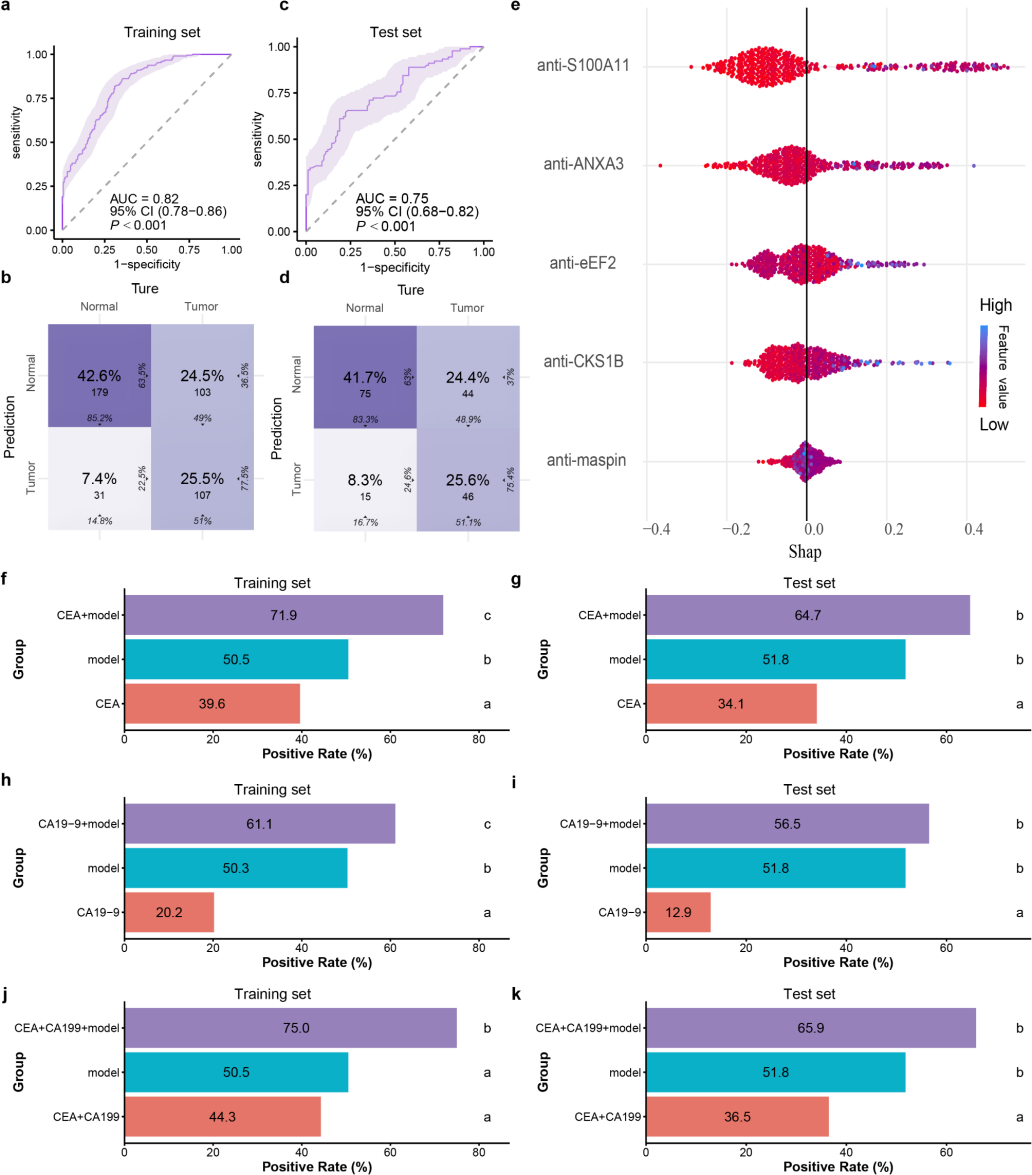
Diagnostic performance of the RF model. **a** ROC curve of the RF model in the training set. **b** Confusion Matrix of the RF model in the training set. **c** ROC curve of the RF model in the test set. **d** Confusion Matrix of the RF model in the test set. **e** Attributes of characteristics in SHAP. Each line represents a feature, and the abscissa is the SHAP value. Blue dots represent higher eigenvalues, and red dots represent lower eigenvalues. **f** Bar chart of positive rate in the training set. **g** Bar chart of positive rate in the test set. **h** Bar chart of positive rate in the training set. **i** Bar chart of positive rate in the test set. **j** Bar chart of positive rate in the training set. **k** Bar chart of positive rate in the test set

The RF model exhibited consistent performance across training and test sets. It achieved an AUC of 0.82 (95% CI: 0.78–0.86) with 68.10% accuracy in the training set (Fig. 6a and b; Table 4), and an AUC of 0.75 (95% CI:0.68–0.82) with 67.22% accuracy in the test set. (Figure 6c and d; Table 3).

**Table 4:** Subgroup performance of the Five-TAAbs model in clinical diagnostics

Characteristic	Clinical Subgroup	*n*	AUC	95%CI	Sen(%)	Sep(%)	PPV(%)	NPV(%)	ACC(%)	F1	YI	Kappa	*P*
	Training Set	210	0.82	0.78–0.86	50.95	85.24	77.54	63.48	68.10	0.61	0.36	0.36	
**Age**	≥ 50	162	0.84	0.81–0.88	54.94	85.24	74.17	71.03	72.04	0.63	0.40	0.41	0.017
	<50	48	0.75	0.68–0.82	37.50	85.24	36.73	85.65	76.36	0.37	0.23	0.23	
**Gender**	Male	102	0.82	0.77–0.86	50.93	85.24	63.95	77.16	73.58	0.57	0.36	0.38	0.823
	Female	108	0.83	0.78–0.87	50.98	85.24	62.65	78.17	74.04	0.56	0.36	0.38	
**Site**	Colon	73	0.81	0.76–0.87	49.32	85.24	53.73	82.87	75.97	0.51	0.35	0.36	0.855
	Rectum	118	0.82	0.77–0.86	49.15	85.24	65.17	74.90	72.26	0.56	0.34	0.36	
**Stage**	Early	141	0.80	0.75–0.84	43.26	85.24	66.30	69.11	68.38	0.52	0.29	0.30	0.085
	Advanced	11	0.88	0.80–0.97	72.73	85.24	20.51	98.35	84.62	0.32	0.58	0.26	

Sen Sensitivity, Sep Specificity, PPV Positive Predictive Value, NPV Negative predictive value, ACC Accuracy, YI Youden Index

### Interpretation and application of RF model

An online application for this RF model was developed, and SHAP was integrated to enhance the interpretability of the machine learning model, thereby increasing its utility in clinical settings. The bee swarm plot illustrates how the key characteristics in the dataset influence the model’s output, highlighting that anti-S100A11 has the highest SHAP values among all features (Fig. 6e).

To enhance its clinical utility, the RF model was deployed as a user-friendly web application accessible through a Shiny server (https://qzan.shinyapps.io/CRCPred/). Users can input TAAb values for one sample using the web interface, and the predicted likelihood of CRC diagnosis will be returned. The application leverages machine learning algorithms for real-time online computation.

### Enhanced diagnostic performance of the 5-TAAbs immunodiagnostic model combined with CEA and CA19-9

Analysis of patients with CEA and CA19-9 test results demonstrated that combining the RF model with these biomarkers significantly improved the positive rate. In the training set, the RF model’s positive rate ranged from 50.3% to 50.5%, while CEA and CA19-9 achieved rates of 39.6% and 20.2%, respectively (Fig. 6f and h). Combining CEA and CA19-9 increased the positive rate to 44.3%. Notably, incorporating the RF model with CEA and CA19-9 further boosted the positive rate to 75.0%, representing a 30.7% improvement over the combination of CEA and CA19-9 alone (Fig. 6j).

Similar trends were observed in the test set. The RF model achieved a positive rate of 51.8%, while CEA and CA19-9 rates were 34.1% and 12.9%, respectively (Fig. 6g and i). The combination of CEA and CA19-9 yielded a positive rate of 36.5%. When combined with the RF model, the positive rate increased to 65.9%, representing a 29.4% improvement over the CEA and CA19-9 combination (Fig. 6k).

### Subgroup analysis of the 5-TAAbs immunodiagnostic model for clinical application

Due to the limited sample size for certain clinical characteristics, the diagnostic values of the model in subgroups were primarily assessed in the training dataset. A subgroup analysis of clinical features such as age, gender, site, and stage was performed. The focus is on clinical features such as age, gender, lesion location, and disease staging. The findings revealed that the model demonstrated significantly better diagnostic efficacy for individuals aged 50 years and above compared to those under 50 years (*P* = 0.017) (Table 4). CRC diagnosed in individuals under 50 years is considered early-onset, while diagnosis at age 50 and above is termed late-onset CRC. Given the well-established positive correlation between age and the incidence risk of late-onset CRC, the model’s superior diagnostic performance in individuals aged 50 and over suggests its potential for future CRC screening programs targeting this high-risk population. However, the DeLong test revealed no significant difference in model performance between early and advanced stages of the disease (*P* = 0.085).

## Discussion

CRC poses a significant global health burden, emphasizing the critical need for early diagnosis and improved patient outcomes [1]. Multi-omics analysis provided a comprehensive view of molecular changes in CRC, facilitating the identification of antigens from various biological pathways. By integrating single-cell transcriptome data from 74 individuals and proteome data from 197 individuals, this study screened 12 candidate TAAs and identified 5 TAAbs for CRC diagnosis through a two-phase ELISA validation. While the individual AUCs of these five TAAbs ranged from 0.58 to 0.64 in the training set and 0.62 to 0.69 in the test set, their combination within the RF model significantly improved diagnostic performance. Notably, combining the 5-TAAb RF model with established biomarkers like CEA or CA19-9 demonstrated superior performance in CRC detection.

The identification of TAAs is crucial for understanding CRC pathogenesis. Mitochondrial DNA transfer contributes to cancer progression by inducing epithelial cells to produce pro-cancer cytokines [44]. Furthermore, the accumulation of somatic mutations in cancer genomes results in the presence of multiple driver gene mutations within a single tumor [45]. The driver genes cataloged in the IntOGen and OncoKB may serve as potential TAAs [46]. Notably, stage-specific embryonic antigens, often present on both pluripotent stem cells and cancer stem cells, are considered potential diagnostic markers and therapeutic targets [47, 48]. By selecting genes commonly upregulated across different subtypes of CRC, we aimed to ensure broader applicability of the identified candidate genes.

In the training set, the AUC for these five TAAbs ranged from 0.58 to 0.64, while in the test set, the AUC ranged from 0.62 to 0.69. Notably, the diagnostic performance of these TAAbs in CRC has not been confirmed in previous studies. Yang *et al*. employed the SERPA method to identify anti-maspin and anti-ANXA3 as potential biomarkers for colon cancer, demonstrating differential expression patterns in a limited cohort of eight patients with colon adenocarcinoma and liver metastasis at various stages [49]. However, these findings lack validation in larger, population-based studies. Moreover, maspin expression is known to be downregulated during the early stages of tumorigenesis, and CEA may potentially influence its expression levels in CRC [50]. Yusuke *et al*. have determined that CRC patients exhibit significantly higher levels of eEF2 IgG antibodies compared to healthy individuals (*P* < 0.01) [51]. Studies have shown that eEF2 protein levels are significantly elevated in many different cancer types compared to normal tissues [51]. Research indicate that CRC overexpresses S100A11, and S100A11 undergoes nucleocytoplasmic translocation during cancer development, potentially impacting cancer cell proliferation [52]. Moreover, another study shows that serum S100A11 levels in CRC patients are significantly overexpressed [53]. *CKS1B* promotes cell proliferation by regulating the activity of cyclins and cyclin-dependent kinases, playing a key role in controlling the transition from the G1 to S phase of the cell cycle [54].

IgG isotype autoantibodies are abundant and diverse in human serum, and their levels can be influenced by disease conditions, including cancer [55]. An integrative analysis has demonstrated a significant increase in IgG-secreting plasma cells within CRC tissues compared to normal and adjacent tissues [56]. The presence of specific IgG antibodies in plasma offers a valuable resource for clinical detection [55]. Liu *et al.*, after reviewing multiple studies, found that p53 autoantibody levels are significantly elevated in the blood of CRC patients compared to healthy controls. Furthermore, patients with negative serum p53 antibody detection exhibited longer disease-free and overall survival, highlighting its potential as a prognostic biomarker for early detection and clinical prognosis [14]. Study showed that although 69 CRC-associated TAAbs demonstrated high specificity (> 85%), their individual sensitivity was generally low (< 30%) [57, 58]. However, combining multiple autoantibodies significantly enhances sensitivity without compromising specificity, where combined sensitivity increased from 18.1%-35.1% to58.5% [57, 59]. In our study, the sensitivity of individual markers ranged from 20.48% to 36.67% in the training set and from 22.22% to 37.78% in the test set, with specificity exceeding 85% in both sets. Training an RF model using five TAAbs resulted in a sensitivity of 50.95% in the training set and 51.11% in the test set. Notably, combining this RF model with CEA and CA19-9 significantly enhanced diagnostic accuracy for CRC, emphasizing the valuable complementary role of these biomarkers alongside traditional clinical biomarkers.

The application of machine learning algorithms in the field of oncology has significant advantages in terms of accuracy and efficiency [16, 60]. Yin *et al.* developed an extracellular vesicle–related RF model for CRC diagnosis, achieving an AUC of 0.960 [61]. In this study, models based on the decision tree ensemble family, such as RF, LightGBM, and XGBoost, demonstrated superior performance. In comparison, while LR serves as a baseline model, its performance was notably inferior to that of tree-based models. Notably, RF and Stacking models emerged as the top two performers in terms of diagnostic accuracy, showcasing considerable generalization capability on the test set (*P* = 0.204). Therefore, this study selected the simpler RF model, allowing for interpretable results through SHAP.

This study presents several key strengths. Firstly, the integration of multi-omics data and CMS classification enhances the robustness of TAA selection. Secondly, rigorous validation of candidate TAAs ensures the reliability of the identified biomarkers. Thirdly, the application of machine learning algorithms improves diagnostic accuracy. Furthermore, the optimal RF model has been deployed as a Shiny app to enhance practical usability.

However, certain limitations need to be acknowledged. The sample size, while adequate for initial analysis, may limit the power of subgroup analyses. Additionally, the study population was predominantly from central China, which may limit the generalizability of the findings. Furthermore, the impact of potential confounding factors, such as smoking and alcohol consumption, was not assessed. Future large-scale, multi-center studies are warranted to further validate the diagnostic performance of the developed model and its generalizability across diverse populations.

## Conclusions

In conclusion, this study has developed and validated a novel diagnostic method for CRC, utilizing a panel of TAAbs, and implementing a robust machine-learning model. This user-friendly model is accessible through a web application (qzan.shinyapps.io/CRCPred/), offering a promising tool for CRC diagnosis. The optimal model proposed in our study can significantly enhance the diagnostic performance of CEA and CA19-9.

## Electronic supplementary material

Below is the link to the electronic supplementary material.

Supplementary Material 1

Supplementary Material 2

Supplementary Material 3

## Data Availability

The datasets supporting the conclusions of this article are included within the article. All data utilized in this study are accessible from the corresponding authors upon reasonable request.

## Abbreviations

CRC: Colorectal Cancer
TAAs: Tumor-associated Antigens
TAAbs: Tumor-associated Autoantibodies
ELISA: Enzyme-linked Immunosorbent Assays
SERPA: Serological Proteome Analysis
CMS: Consensus Molecular Subtypes
HC: Healthy Control
GEO: Gene Expression Omnibus
CPTAC: Clinical Proteomic Tumor Analysis Consortium
SBA: Small Intestine Carcinoma
GO: Gene Ontology
SBI: Specific Binding Index
OD: Optical Density
QC: Quality Control
RF: Random Forest
PR: Precision-recall
DCA: Decision curve analysis
